# Supplementary material for: Obesity Is Associated with the Severity of Periodontal Inflammation Due to a Specific Signature of Subgingival Microbiota
Source: Int J Mol Sci. 2023 Oct 12;24(20):15123. doi: 10.3390/ijms242015123 (PMC10606428; doi:10.3390/ijms242015123)
Supplement: Supplementary file 1 [file ijms-24-15123-s001.zip › ijms-2635015-supplementary.pdf]

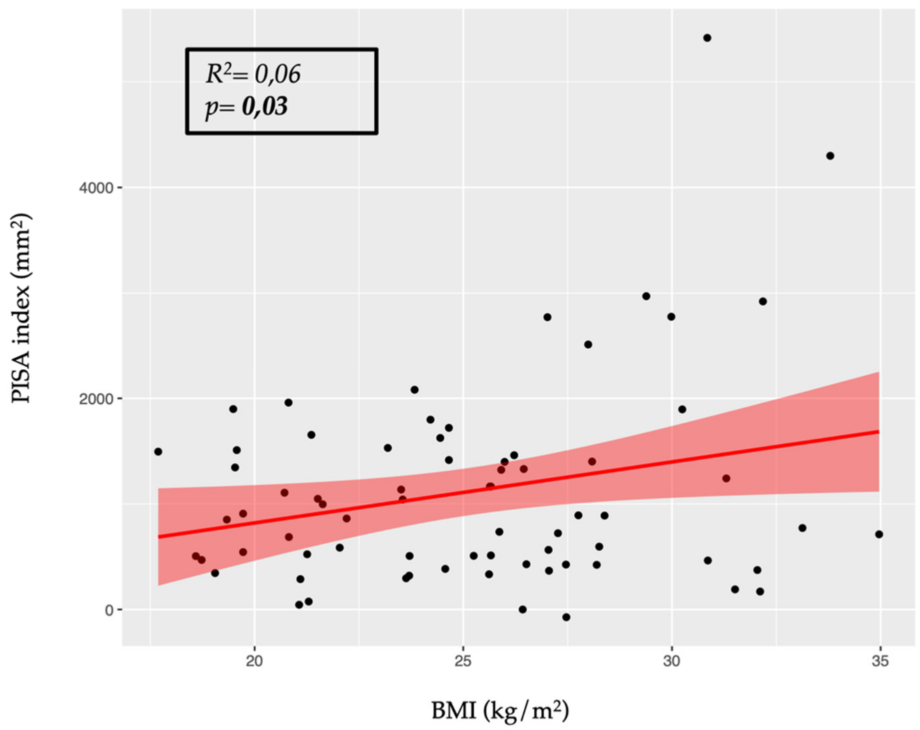

Figure S1. Pearson's correlation analysis between PISA index and BMI. We observed a correlation between local inflammatory surface, represented by the PISA index, and BMI. In order to avoid two distinct groups, we took advantage of the epidemiological data of overweight patients to obtain the distribution of local inflammation depending on BMI.

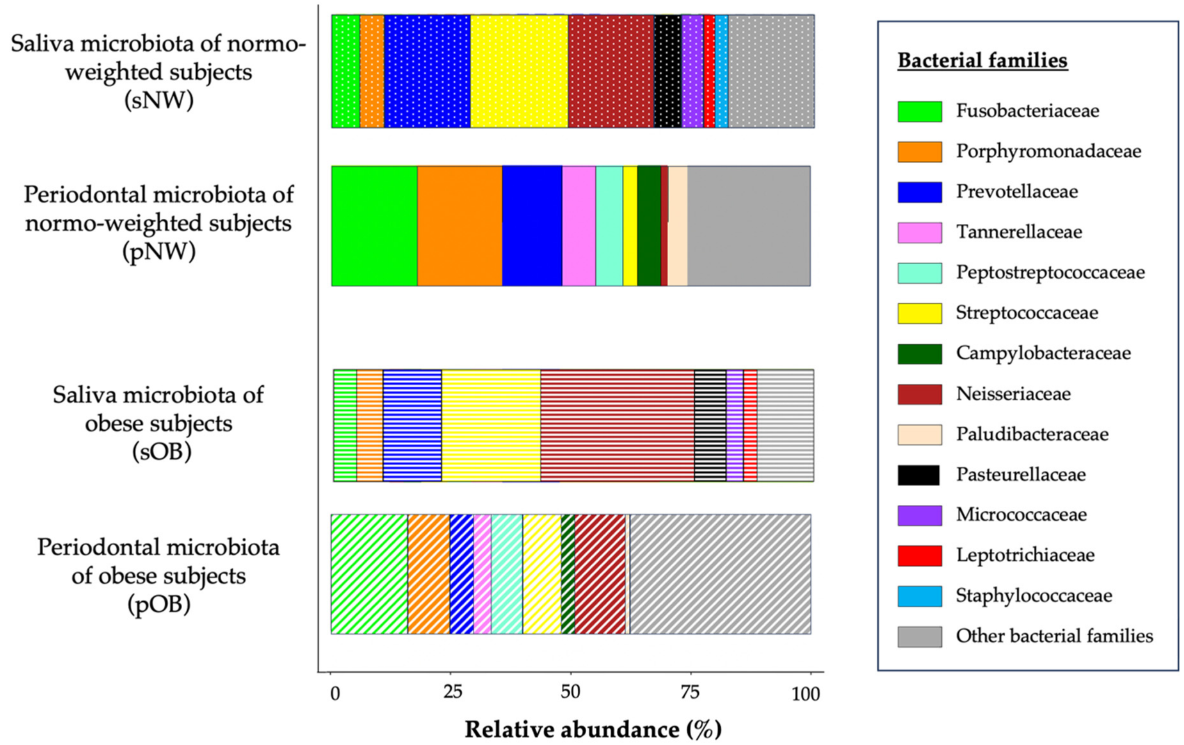

Figure S2. Top 10 relative abundances (%) for taxonomic families in salivary and periodontal microbiota of obese and normo-weighted subjects.

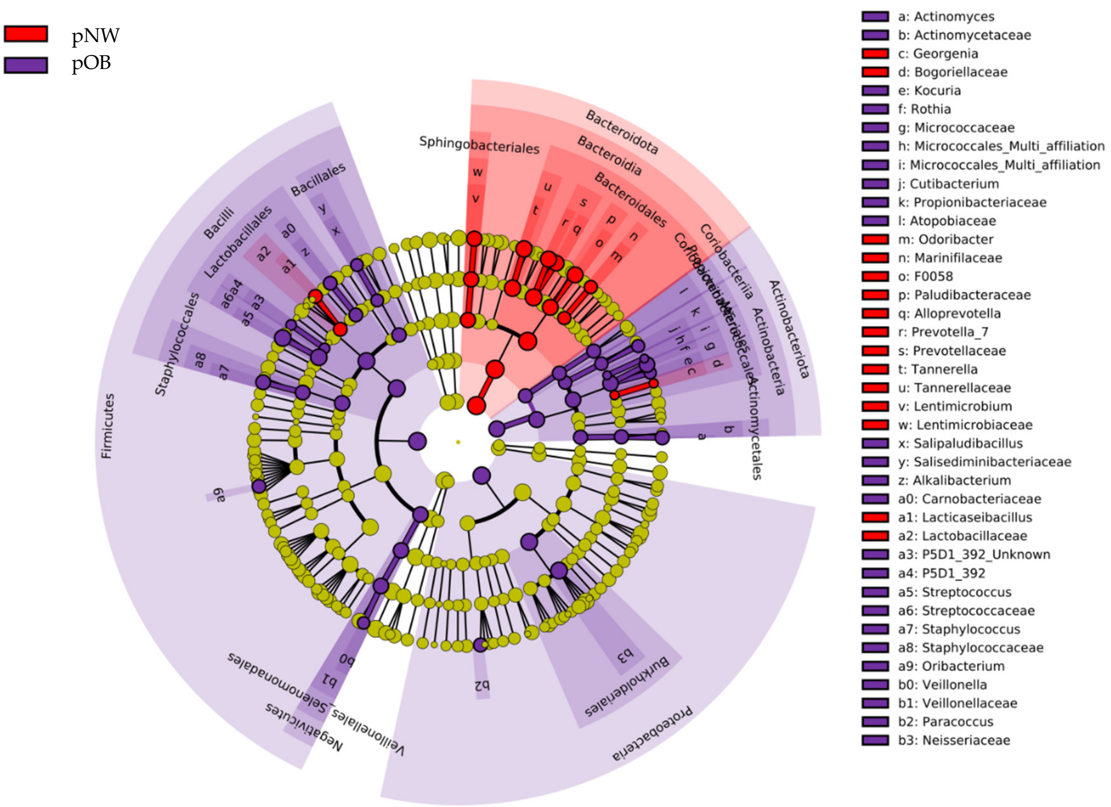

Figure S3. Linear discriminant analysis effect size (LEfSe) analysis-based cladogram for periodontal microbiota of obese and normo-weighted subjects

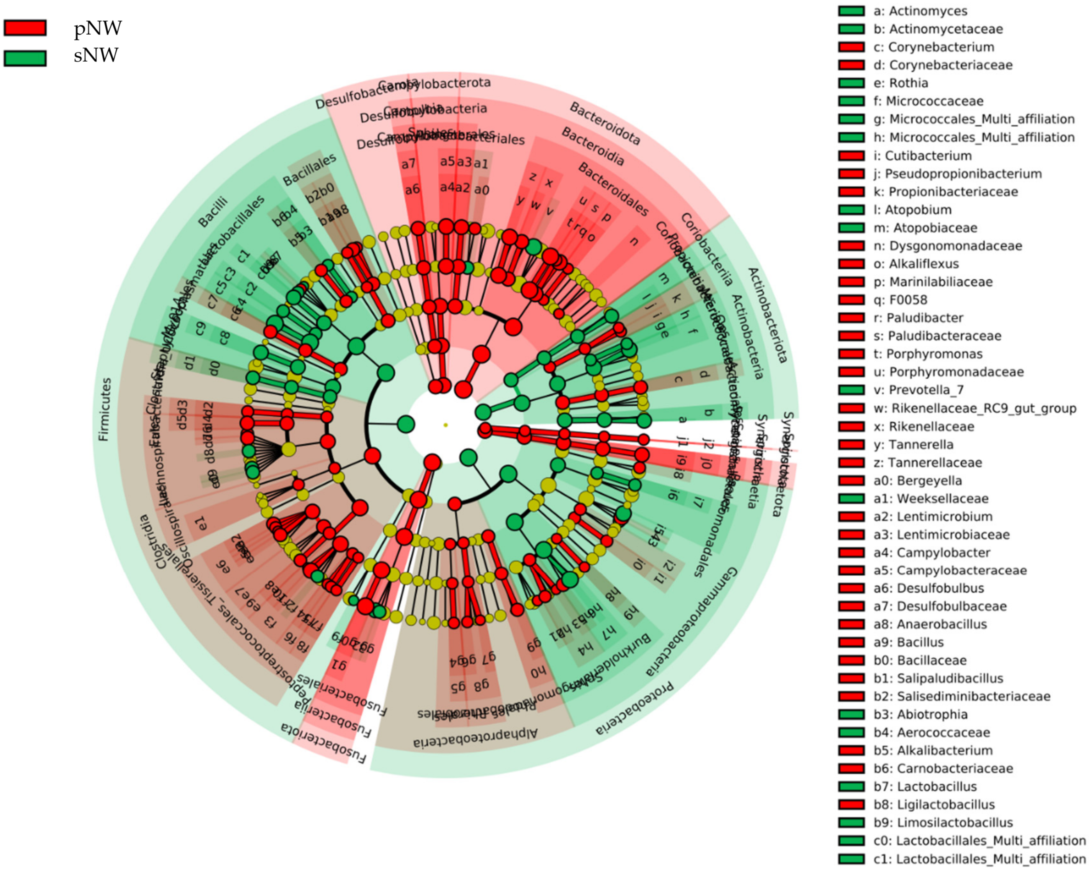

Figure S4. Linear discriminant analysis effect size (LEfSe) analysis-based cladogram of periodontal and salivary microbiota in normo-weighted subjects.

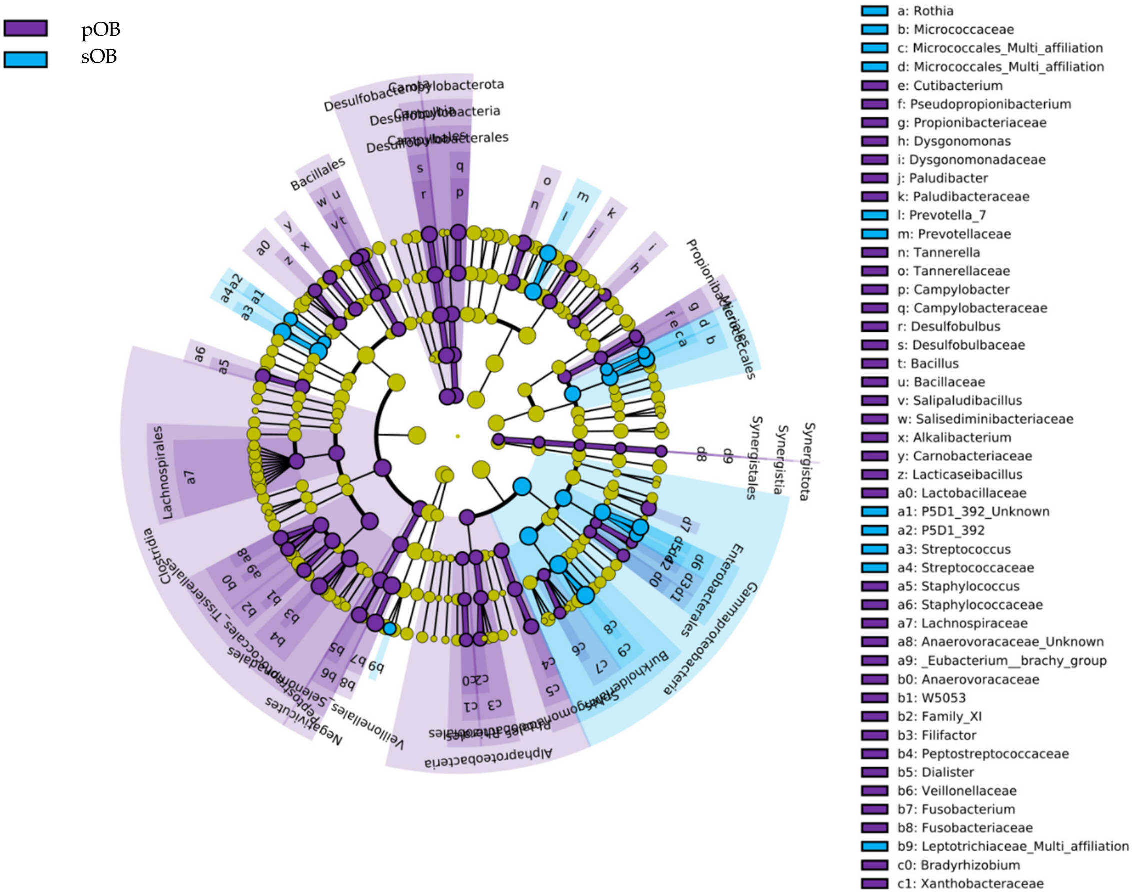

Figure S5. Linear discriminant analysis effect size (LEfSe) analysis-based cladogram of periodontal and salivary microbiota in obese subjects.

	Relative abundance of Staphylococcaceae				p-value
	Periodontal microbiota		Salivary microbiota		
	pNW (n = 34)	pOB (n = 11)	sNW (n = 34)	sOB (n = 11)	
	0	5,54917240202491	0	0	
	0	2,20167958748987	0	0,04770739	
	0,0017690974065032	1,30684174153421	0	0	
	0,0059349529160402	0,0507283136459164	42,4307036	0	
	0,00132329394328362	0,178422205659823	0	0	
	0	2,12809286223399	0	0	
	1,68074563988388	0,953972020984262	0	0	
	0,000772105376941845	0,113687659873272	0	0	
	1,7507311405793	0	11,1088317	0	
	0,625988490317779	0	20,4362078	0,00525127	
	0	0	0		
	0,003180358108323		0		
	0,00135987815491732		0		
	0		0		
	0		0		
	0		0		
	0		0		
	0,0873871249635887		0		
	0		0		
	11,5166551030563		0		
	0,792546967790361		0		
	0		0		
	0		0		
	0		20,4362078<br>0		
	0,00177604120415594		0		
	0,0039714058776807		0		
	0		0		
	0		0		
	0		0		
	0		0,00549843		
	0		0		
	0		15,423589		
	0		0		
	0		0		
**Mean ± SD**	0,484533576 ± 1,996730174	1,134781527 ± 1,693212834	2,629695849 ± 8,428264644	0,004814424 ± 0,014312952	**pNW vs. pOB * p = 0,006**
**Ratio Perio/Saliva**	**-4,427272695**	**0,9957574**			

Table S1. Relative abundance of Staphylococcaceae in periodontal microbiota.

	Relative abundance of Flavobacteriaceae				p-value
	Periodontal microbiota		Salivary microbiota		
	pNW (n = 34)	pOB (n = 11)	sNW (n = 34)	sOB (n = 11)	
	0,000777157778572206	0	0,260704692684468	1,45	
	0,308523562887653	0	0,730273141122914	0,0106016432547045	
	12,5004422743516	2,28956461644782	0,246347751360642	1,09434895511314	
	1,29381973569676	0,0710196391042829	0,0147048011175649	0,924304339365635	
	0,919689290582117	0,185197732457032	0,212862026704509	0,247912317327766	
	0,087830584561335	3,50269829956216	0,281728853157425	0	
	0,178988496714906	0,0780664501623782	0,207845044885685	6,58859470468432	
	6,56289570400568	2,12328423586846	0,176645593157307	3,20090572251955	
	0,0480749969953127	5,36531813510231	0	1,11051502145923	
	0	0,425408996612484	0	3,05624113847608	
	1,23046711790758	1,61754855994642	1,2126627521062	0,141168878312427	
	7,69964698024998		0,578679125079635		
	0,0720735422106179		1,01419878296146		
	0,152296885684093		0,028169014084507		
	0		0,313686643552914		
	0,36246476037052		2,24211629970159		
	2,39599276471407		3,19783573806881		
	0,0606855034469366		0		
	1,56167045648058		0,495635449030922		
	0		0,8		
	0		0		
	0,20995597697257		0,978574371652246		
	1,41497045305319		0,342625759227535		
	0,234279823821573		0,420066631258751		
	0,43690613622236		12,1785894347656		
	0,631453534551231		0,0558174150334904		
	0,062439295129735		1,34044393196887		
	5,31077024756974		0,257450460290217		
	0,301309905166672		0,789714450811383		
	0,0753078763178878		0,197943586077968		
	0,180813318777293		2,01454659620114		
	0,607889442840899		1,37857900318134		
	0,682790391576176		0,53194210014833		
	2,84802739086452		1,26008778139601		
**Mean ± SD**	1,467674352 ± 2,73873931	1,423464242 ± 1,770878136	0,992955213 ± 2,105828083	1,62041752 ± 1,989519754	**pNW vs. pOB p = 0,8307**
**Ratio Perio/Saliva**	**0,323449911**	**-0,138361943**			

Table S2. Relative abundance of Flavobacteriaceae in periodontal microbiota.
